# Fever and Ague Destroyer

**Published:** 1874-02

**Authors:** 


### FEVER AND AGUE DESTROYER.

It has been observed that a thick hedge of sunflower growing around a dwelling, is a great protection to the family from fever and ague in localities where it abounds. F. S. Olmstead, the beautifier of the Central Park, writes to the *New York World*, Dec. 16th, 1873, of the eucalyptus tree, now growing from the seed in the green house of the park, that the value of the tree in abating malarious influences is considerable. The word malarious means simply "bad air;" made bad from any cause; he intended to mean that kind of bad air which promotes disease, especially fever and ague in flat waters and luxuriant localities. This agent is more fitly named miasm. So the phrase "miasmatic influence" should have been used. The literal meaning of miasm is "emanation," a rising from, as it seems to come up out of the earth, as a product of vegetable decomposition from the continued action of warmth and moisture; but whether this miasm is of a vegetable or animal character has not as yet been demonstrated. It was taught in medical schools forty years ago that any vegetable growth antagonized miasmatic influences, its efficiency being in proportion to the luxuriousness of the growth. The sun flower grows with amazing rapidity in rich soils, as in the bottom lands of rivers, hence its efficiency in the direction named was so very striking as to cause the impression, that there was some peculiar element in the plant itself which antagonized miasmatic influences. Mr. Olmsstead very truly remarks that the influence of all trees on climate and health is more decided than is generally supposed. We know that low lands, well drained, become more healthy and more productive, and it may be that rank vegetation consumes or absorbs, and then evaporates and sends into the higher air above us, so much of the water as to answer the purpose of drain pipes. The experiments of Pettenkofer at Munich, show that a single oak tree, discharged through its leaves during one summer, eight times and a half as much water as fell in rain on the surface which it covered. The real practical lesson taught by these facts is simply this: dwellings now unhealthy may be made salubrious by a well arranged system of drainage of all the surface within half a mile of it. It is certain that districts where fever and ague abounded have become healthy when a large growth of luxurious trees has taken place. The encalyptus is said to be more luxuriant in productive soils and temperatures than even the sun flower or the ailanthus. Whether it would thrive out doors in the Northern States has not been ascertained. The acknowledged fact that the sun flower hedge at a distance of fifty feet from a dwelling in fever and ague districts exempts the family from the malady, is not because there is any peculiar virtue in the sun flower plant itself, but because it grows with great rapidity, consumes a vast amount of water in the support of its growth and thus antagonizes the generation of miasm, the thing which causes all forms of autumnal diseases, inducing every grade of fever.

